# The influence of culture and religion on the self-management of type 2 diabetes in South Asian migrants: a systematic review

**DOI:** 10.1186/s12889-026-27776-5

**Published:** 2026-06-05

**Authors:** Navodya C. Selvaratnam, Fung Kuen Koo, Kanchana Ekanayake, Amit Arora

**Affiliations:** 1 School of Medicine, Faculty of Health, Western Sydney University, Campbelltown Campus, Locked Bag 1797, Penrith, NSW 2751 Australia; 2 Postgraduate Institute of Medical Sciences, University of Peradeniya, Getambe, Peradeniya, Sri Lanka; 3 Health Equity across Lifespan (HEAL) Research Lab, Campbelltown, NSW 2560 Australia; 4 School of Nursing and Midwifery, Faculty of Health, Western Sydney University, Hawkesbury Campus, Locked Bag 1797, Penrith, NSW 2751 Australia; 5 Susan Wakil School of Nursing, Faculty of Medicine and Health, The University of Sydney, Camperdown, NSW 2006 Australia; 6 University of Sydney Library, The University of Sydney, Camperdown, NSW 2006 Australia; 7 Translational Health Research Institute, Western Sydney University, Campbelltown, NSW 2560 Australia; 8 Young and Resilient Research Centre, Locked Bag 1798, Penrith, NSW 2751 Australia; 9 Discipline of Child and Adolescent Health, Faculty of Medicine and Health, The Children’s Hospital at Westmead Clinical School, The University of Sydney, Westmead, NSW 2145 Australia; 10 Oral Health Services, Sydney Local Health District and Sydney Dental Hospital, NSW Health, Surry Hills, NSW 2010 Australia; 11 Aureolin Research, Consultancy & Expertise Development (ARCED) Foundation, 7 Avenue 5, Dhaka 1216 Bangladesh

**Keywords:** Type 2 diabetes, Self-management, South Asian migrants, Culture, Religion

## Abstract

**Background:**

Despite the high prevalence of type 2 diabetes (T2D), research exploring the inter-relationship of diabetes self-management, culture and religion in South Asian migrants is minimal. This systematic review aimed to investigate the influence of cultural and religious beliefs and practices on the self-management behaviours of South Asian migrants with T2D.

**Methods:**

A mixed-methods systematic review was conducted. Five electronic databases, Medline (Ovid), Embase (Ovid), Cumulative Index to Nursing and Allied Health Literature (CINAHL) (EBSCO), Scopus, and Web of Science (Clarivate), were searched systematically using predetermined inclusion criteria. Two reviewers independently assessed the full texts of the articles retrieved. The methodological quality of articles was assessed using the Mixed Methods Appraisal Tool. Data synthesis was guided by thematic analysis.

**Results:**

A total of 22 studies (18 qualitative and four mixed-methods) were included in this systematic review. Five themes are presented: (1) Adapting to diabetes: impacts on food habits and dietary choices; (2) Diabetes threatens cultural conventions and identity; (3) Religious beliefs, practices, and prohibitions complicate diabetes management; (4) Social support and awareness to promote self-care; and (5) Individuals seek multiple sources for diabetes management.

**Conclusion:**

Numerous complexities surround South Asian migrants with T2D and impact their self-management behaviours. Understanding these complexities could facilitate improved patient care and health outcomes for this population.

**Supplementary Information:**

The online version contains supplementary material available at https://doi.org/10.1186/s12889-026-27776-5.

## Introduction

South Asian migrants are one of the largest foreign-born populations in Western countries [1] and have a recorded higher type 2 diabetes (T2D) prevalence than host populations and other Asian migrants [2, 3]. T2D develops due to impaired functioning of pancreatic beta cells and insulin resistance [4, 5]. Genetic predisposition, family history, obesity, unhealthy dietary habits, low physical activity, ethnicity, age and low socioeconomic status are among the factors attributed to a higher risk of T2D [6, 7]. T2D is associated with complications such as cardiovascular disease, retinopathy, renal failure, amputation, and consequent morbidity and mortality [8].

Globally in 2021, one in ten adults aged 20–79 years, that is 537 million individuals, were living with diabetes. This number is estimated to increase to 643 million by 2030 [9]. However, 81% of the global adult population with diabetes lives in low and middle-income countries [9]. In 2021, lower-middle-income countries such as India [10] had the second-highest T2D population in the world, while Pakistan [11] and Bangladesh [12] shared some of the highest burdens of T2D [9]. South Asians in particular experience an increased risk of cardiometabolic disorders such as T2D [13]. South Asians include people from India, Pakistan, Bangladesh, Sri Lanka, Nepal, Bhutan, the Maldives, and Afghanistan [14]. In search of better opportunities or governed by humanitarian reasons, millions of South Asians migrate. They make up about 3% of the region’s working-age population, of which a quarter resides in high-income countries such as the United States of America (USA), Canada, the United Kingdom (UK), and Australia [15–18].

Research highlights the principle of a healthy immigrant effect, that is, individuals who choose to migrate have better health than the native population, which may deteriorate the longer they stay in the host country [19]. For instance, a higher number of chronic health conditions is predicted at younger ages, particularly at ages 55–64, for Asian migrants, compared to native Europeans [20]. In the USA, the prevalence of chronic health conditions, such as diabetes, among migrants is observed to increase with increasing length of residence, regardless of age at migration [21]. Compared to their host-country counterparts, South Asians have shown a 5–10 year earlier risk of T2D development [13, 22]. They are also observed to have a higher prevalence of T2D and associated complications [15, 23, 24]. For example, compared to the Canadian population, a 1.18% to 2.25% higher prevalence of T2D was observed in South Asian migrants [25]. Thus, it creates a spectrum of significant physical, mental, social, and economic burdens [26, 27]. 

T2D-associated complications can largely be prevented through effective self-management practices [4, 28], including self-monitoring of blood glucose levels, dietary changes, physical activity, medication adherence, and regular eye and foot checks. Previous research has identified that cultural and religious beliefs and practices could affect self-management behaviours [4, 28–30]. As highlighted by Fleming and Gillibrand [31], it is imperative to be cautious not to ascribe the observed higher prevalence of T2D in South Asian migrants to their ethnic and cultural influences. Their beliefs and practices may vary depending on varying factors such as context, socioeconomic conditions, family influences and past experiences [31]. They may share certain cultural norms and social values [32]. However, South Asian migrants are highly diverse in all facets of ethnicity, language, culture, and religion [4, 33]. For example, they speak several languages, including Hindi, Tamil, Urdu, Punjabi and Sinhala, and observe different religions that can significantly influence their health behaviours, including Hinduism, Islam, Buddhism, and Sikhism [29, 34, 35]. More importantly, the vast majority adhere to a dharmic faith, and their religious and spiritual beliefs and practices are often embedded in daily life, though practised differently [34, 35].

### Purpose of the study

A holistic view of health, considering psychosocial, spiritual and physical dimensions alongside the biomedical, is essential for all populations. However, this is particularly important for migrants, such as South Asian migrants, who often face cultural and social challenges [13]. Cultural beliefs and practices influence individual preferences, behaviours and healthcare utilisation [36]. Religiosity and spirituality similarly influence mental health and disease outcomes, though they are often overlooked [13]. A deeper understanding is therefore needed to design, develop and disseminate targeted measures to prevent and manage cardiometabolic disorders such as T2D in South Asian migrants [36]. Research exploring the inter-relationship of diabetes self-management, culture and religion in South Asian migrants remains limited [37, 38]. A few systematic reviews have explored cultural influences on self-management of T2D among South Asian migrants [29, 31, 39, 40]; however, these studies mainly focus on migrants from India, Pakistan, Bangladesh and Nepal, narrowing the spectrum of South Asian migrants. One study also included Malaysian-Indian migrants as South Asian [40], though they might better represent Southeast Asian [41]. While Patel et al. [29] offered recent insights, their scope was confined to qualitative studies within the UK, with most primary studies being comparatively old.

Existing evidence acknowledges that social, cultural, religious and structural beliefs, low health literacy levels, and gender and language-specific barriers influence T2D self-management among migrants [42–44]. When structured interventions are tailored to minority ethnic groups with diabetes, integrating elements of culture, religion, language and health literacy can improve their health outcomes [45, 46]. Therefore, it is vital to identify and understand these elements in order to design and implement culturally appropriate interventions to support diabetes self-management and reduce the diabetes burden on South Asian migrants in a host country. Healthcare professionals should also reflect on the migrant populations they serve, recognising how culture and religion influence health beliefs and behaviours, and adapt healthcare accordingly [46].

By including diverse qualitative, quantitative and mixed-methods studies with more recent evidence, this systematic review aims to provide insights into the cultural and religious beliefs and practices of South Asian migrants and how these shape their diabetes management. It also seeks to identify opportunities for healthcare professionals, governments and policymakers to intervene, better supporting South Asian migrants in managing their condition and reducing the diabetes burden, particularly given the increasing T2D prevalence, the growing demand for equity in healthcare provision, and the limitations of prior interventions in adequately addressing the elements of culture and religion in this population.

### Objectives of the study

The primary objective of the present review is to investigate and explore how culture and religion influence the self-management behaviours of South Asian migrants with T2D. Additionally, this study intends to inform healthcare providers and health promotion interventions about how pivotal culture and religion are in the lives of South Asians, especially in diabetes self-management. Lastly, we aim to contribute to the healthcare systems’ efforts to provide culturally sensitive and appropriate healthcare services to culturally and linguistically diverse communities to mitigate health disparities and poor health outcomes.

## Methods

A mixed-methods systematic review, incorporating qualitative and mixed-methods studies, was conducted to explore the influence of cultural and religious beliefs and practices on the self-management of T2D in South Asian migrants. The protocol for this study was registered in the PROSPERO International Prospective Register of Systematic Reviews (CRD42023414483) [47]. The present study followed Preferred Reporting Items for Systematic Reviews and Meta-Analyses (PRISMA) guidelines for reporting the review process and findings [48].

### Inclusion and exclusion criteria

The present systematic review initially focuses on broader cultural and religious influences, subsequently narrowing its focus to T2D self-management behaviours in the sub-population of South Asian migrants. Hence, (1) studies that explore the influence of culture and religion on the self-management of T2D, (2) studies focused on South Asian migrants, (3) peer-reviewed studies published in the English language and available in full text, and (4) studies with a qualitative, quantitative, or mixed-methods study design were considered for inclusion. The dates of publication were not restricted, and thus, studies from the date of inception of the database until 8^th^April 2026 were considered for inclusion.

 Studies that focused only on type 1 diabetes (T1D) and/or gestational diabetes were excluded as they were beyond the scope of the review. Studies based on South Asians residing in their home countries were also excluded, as self-management behaviours, factors that influence these behaviours, and their extent of influence may differ between South Asian migrants and their counterparts residing in their home countries. Studies published in languages other than English were excluded due to the financial considerations and time constraints involved in translation. Reviews, conference abstracts, reports, perspectives and grey literature were also excluded, as the objective of the present study is to explore the understudied inter-relationship of T2D self-management, culture and religion in South Asian migrants rather than provide an exhaustive list of evidence.

### Search strategy and data sources

In consultation with a health science librarian (KE), an initial search strategy was developed for the Medline (Ovid) electronic database (See Supplementary Material 1). Medical subject headings (MeSH), free-text words, Boolean operators, and syntaxes such as truncation, wild cards, and nested proximity were incorporated in developing the search strategy. Search terms included T2D, self-management, diet, medication adherence, blood glucose monitoring and South Asian migrants. The search terms were identified and developed through pilot testing undertaken on Medline (Ovid). The search strategy developed for Medline (Ovid) was subsequently adapted to the requirements of each electronic database selected for the systematic review (See Supplementary Material 1), including Embase (Ovid), Cumulative Index to Nursing and Allied Health Literature (CINAHL) (EBSCO), Scopus, and Web of Science (Clarivate). The focus was restricted to these five databases as they provide a comprehensive, relevant and quality coverage of both medical and social aspects of the present review. Finally, a systematic literature search was conducted on these databases to identify articles relevant to achieving the review's purpose. The initial search was conducted on 19th March 2023 and subsequently updated on 8^th^ April 2026.

### Study selection

**Fig. 1:**
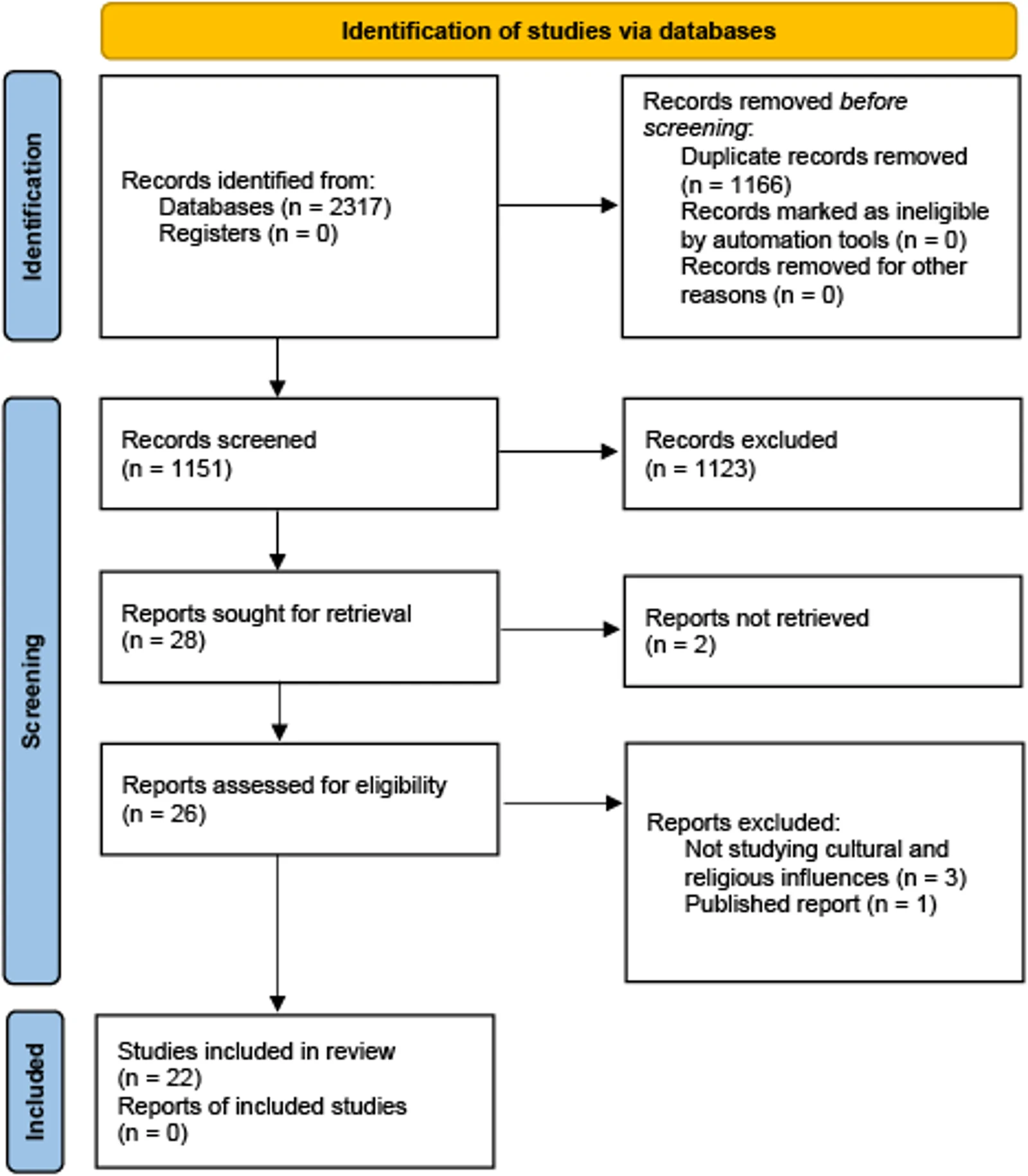
PRISMA flow diagram of study selection and study inclusion

Studies identified through the systematic literature search were initially imported to EndNote 20 reference management software [49] by creating separate EndNote libraries for each database. Subsequently, each database library was imported into the Covidence systematic review software [50]. Duplicates were removed through software and manual allocation. Titles and abstracts were screened against the inclusion and exclusion criteria by two reviewers (NCS and AA) independently. Studies that met the inclusion criteria were retrieved for full-text screening and further assessment. Two reviewers (NCS and AA or FKK) independently assessed each retrieved article against the inclusion and exclusion criteria to consider for inclusion in the systematic review. Any disagreements were resolved through discussion among the three reviewers and consensus-based decisions. The study selection process is presented in a PRISMA flow diagram (Figure 1) [48].

### Methodological quality assessment

The Mixed Methods Appraisal Tool (MMAT) Version 2018 by Hong et al. [51] (See Supplementary Material 2) was utilised to assess the risk of bias or the methodological quality of all included studies. The MMAT facilitates the assessment of the methodological quality of studies with diverse study designs, including mixed-methods study designs, which is often not observed in other tools [52, 53]. Two reviewers (NCS and AA or FKK) performed the quality assessment independently. Any disagreements were resolved through discussion among the three reviewers and consensus-based decisions.

### Data extraction

A simple data extraction form, which was adapted from standard data extraction templates for qualitative and mixed-methods studies, was utilised to extract data from each included study. Three reviewers (NCS, AA and FKK) performed a calibration exercise to ensure consistency across the reviewers. Two reviewers (NCS and AA or FKK) extracted data independently from each included study, and any disagreements were resolved through discussion among the reviewers and consensus-based decisions. The data extracted included the first author, year of publication, country, study aims, race/ethnicity of study participants, sample size, age of participants, gender of participants, study design, methods and key findings.

### Data synthesis

The thematic synthesis method guided data synthesis for the present systematic review, as the included studies were primarily qualitative [54]. Quantitative synthesis methods were not incorporated as quantitative studies were absent among the included studies. In mixed-methods studies, tabular data were translated into sentences to facilitate data synthesis. Accordingly, data under ‘Results’ or ‘Findings’ from all included qualitative and mixed-methods studies were uploaded to Quirkos 2.5.2 computer software for qualitative data analysis [55]. First, the results or findings of all included studies were coded line-by-line. Then the generated codes were grouped to create descriptive themes, and finally, descriptive themes were grouped and refined to develop analytical themes.

## Results

A total of 2317 records were obtained from all database searches. These included n = 376 from Medline (Ovid), n = 779 from Embase (Ovid), n = 188 from CINAHL (EBSCO), n = 470 from Scopus and n = 504 from Web of Science (Clarivate). Duplicate records were removed both automatically by the software and by manual allocation based on the title and authors. After removing duplicates (n = 1166), the search provided 1151 articles for title and abstract screening, which yielded 28 articles for full-text screening. A total of n = 1123 studies were excluded during the title and abstract screening as they failed to satisfy the inclusion criteria. From the full-text screening, a further six studies [56–61] were excluded (See Supplementary Material 3 for more details). This is because, though the six studies appeared to satisfy the inclusion criteria during the title and abstract screening stage, they failed to satisfy the inclusion criteria for the review upon full-text assessment. Finally, 22 studies were included (Figure 1) in the present systematic review. Table 1 summarises the characteristics of all included studies.

**Table 1:** Study characteristics of included studies

Authors and year	Study country	Study aims	Race/ ethnicity	Sample size	Age range (years)	Gender	Study design/ method	Key findings
Abuelmagd et al. (2018)	Norway	To assess how Pakistani women with T2D live with the disease and their response to lifestyle and medical information	Pakistani	120	29–80	All female	Mixed methods. Individual structured interviews	Religious beliefs’ influence on drug intake. A majority reported an unhealthy diet and lack of physical activity; one in four altered drug intake due to religious fasting
Ahmad et al. (2021)	Australia	To investigate medication-taking behaviour and factors influencing medication adherence among Indian migrant patients	Indian	23	33–72	18 males, 5 females	Qualitative. Semi-structured interviews	Negative beliefs about medicine; fear of injection, discomfort, and pain; fear of medication side-effects and dependency; diabetes-related stigma; GP recommendations; medication awareness
Ahmad et al. (2022)	Australia	To explore the religious beliefs and its influence on diabetes self-management in Indian migrants in Australia	Indian	23	18–61 and over	18 males, 5 females	Qualitative. Semi-structured interviews	Belief in god and prayers; religious fasting; seeking help from religious leaders; meditation and yoga benefits; beliefs about insulin and other medication
Akhtar et al. (2024)	New Zealand	To explore medication adherence practices of South Asians prescribed with medications for T2D, and/or hypertension, and/or dyslipidaemia.	Indian, Fijian Indian, Pakistani, Nepalese, Sri Lankan	21	35 – 65	10 males, 11 females	Qualitative. Semi-structured interviews	Fear of medication side-effects and dependency; natural, complementary or alternative medicines use; role of healthcare providers
Choudhury et al. (2009)	United Kingdom	To examine the understanding and beliefs of people with diabetes from the Bangladeshi community living in the UK.	Bangladeshi	14	26–67	4 males, 10 females	Qualitative. Semi-structured interviews	Cause of diabetes; physical activity; information from healthcare professionals, role of family and friends; use of traditional medication; diabetes education
Fagerli et al. (2005)	Norway	To explore how ethnic minority people with diabetes experience dietary advice provided by Norwegian health workers, their strategies in response to the advice, and how they explain their actions	Pakistani	15	38–66	4 males, 11 females	Qualitative. Semi-structured interviews	Struggles of grasping the meaning of advice; lack of food tradition acknowledgement; minority situation; cultural miscommunication
Iqbal et al. (2023)	United Kingdom	To explore T2DM-related beliefs about healthy eating and food practices among British Pakistanis to understand the challenges face in implementing healthy diets	Pakistani	26	18 – 71	12 males, 14 females	Qualitative. Semi-structured interviews	Misconceptions about healthy eating; challenges to adopting dietary changes; significance of traditional food; stigma
Jamil et al. (2022)	United States	To explore how cultural perspectives of South Asian migrants in the US impact medication adherence for T2D and CVD	Pakistani, Indian, Bangladeshi	12	49 – 75	6 males, 6 females	Qualitative. Semi-structured interviews	Self-management and autonomy; religious/spiritual beliefs and medication adherence; complementary and alternative medicines use
Khan et al. (2008)	United Kingdom	To determine the prevalence and reasons for refusal to commence insulin in Bangladeshi patients with T2D	Bangladeshi	212	32 – 77	112 males, 100 females	Mixed methods. Survey. Focus groups	Disease severity, social stigma; needle anxiety; loss of control; lack of perception - benefits and fatalism. A majority agreed to insulin immediately; around one-fifth refused insulin despite repeated counselling
Lawton et al. (2005)	United Kingdom	To explore British Pakistani and British Indian patients' perceptions and experiences of taking oral hypoglycaemic agents (OHAs)	Pakistani and Indian	32	30 – 71 or over	15 males, 17 females	Observational cross-sectional. In-depth interviews	Initial reactions to taking OHAs; perception towards OHAs; self-regulation of OHAs; self-monitoring; reducing food intake
Lawton et al. (2008)	United Kingdom	To explore perceptions on food and eating practices, barriers and facilitators for dietary change, and underpinning social and cultural factors among Pakistani and Indian migrants living with T2D	Pakistani and Indian	32	33 – 71	15 males, 17 females	Grounded theory. In-depth interviews	Information from healthcare providers; South Asian food perception; sharing, and commensality; food strategies
Macaden & Clarke (2006)	United Kingdom	To report on the experiences of South Asians with diabetes in the UK in relation to socio-cultural and dietary practices, religion, and ageing influences on the perception and understanding of risks.	South Asian (Bangladeshi, Indian, and Pakistani)	20	Older	Male and female	Grounded theory. Individual interviews and focus groups	Importance of religious beliefs; fatalism; importance of food and family dynamics
Majeed-Ariss et al. (2015)	United Kingdom	To explore the effects of T2D on the identity of British-Pakistani women and its relationship with self-management	Pakistani	15	31 – 76	All female	Qualitative. Semi-structured interviews	Social support; perceived change in self; barriers to self-management; role re-alignment for successful self-management
Mu’Min Chowdhury et al. (2000)	United Kingdom	To explore food beliefs and classification system of British-Bangladeshis	Bangladeshi	40	24 – 78	20 males, 20 females	Qualitative. Narrative interviews	Importance of religious prohibitions; ethnic dietary customs; diet post-immigration; classification of food; role of cooking; food, health, and illness relationship
Mygind et al. (2013)	Denmark	To explore the perspectives of Pakistani background people with T2D and at least one other chronic condition on medicine use, reasons for fasting, and experiences with counselling during Ramadan	Pakistani	6	42 – 69	1 male, 5 females	Hermaneutics. Semi-structured interviews and medication reviews.	Medicine use during Ramadan; Ramadan fasting; physiological, social, and religious aspects of improved wellbeing
Naeem (2003)	United Kingdom	To explore the current experience and attitude towards diabetes control among Kashmiri men with diabetes in Leeds	Kashmiri	106	Below 40 – over 60	All men	Mixed methods. Survey. Individual interviews	Cause of diabetes; food perceptions. Around one third believed diabetes was due to Allah's will; a majority fasted during Ramadan
Patel et al. (2015)	United Kingdom	To explore attitudes towards accepting insulin therapy among people with T2D in an ethnically diverse population	South Asian (mainly Indian) and White British	18	Over 18	9 males, 9 females	Grounded theory. In-depth semi-structured interviews	Beliefs and concerns about insulin and its necessity; influences on beliefs; negative views from community
Patel et al. (2016)	United Kingdom	To explore the role of social networks and beliefs about diabetes in British South Asians, to better understand their management behaviours whilst holidaying in the East	Indian, Pakistani, Bangladeshi, Nepalese	44	32 – 84	23 males, 21 females	Qualitative. Semi-structured interviews	Differing roles and social support from networks "back home"; diet and diabetes management beliefs; perceived limited role of GP
Timsina et al. (2022)	United States	To understand factors affecting diabetes medication adherence in Bhutanese refugees	Bhutanese	22	19 – 79	10 males, 12 females	Qualitative. Focus groups	Barriers to engage in care; family and community support; culturally contextual person-centred care
Uppal et al. (2016)	Canada	To describe ethnocultural influences associated with T2D management in older Sikh immigrants in Toronto, Canada	Indian	9	44 – 70	5 males, 4 females	Phenomenology. Semi-structured interviews	Social constructions; personal understanding of diabetes management; actualised experiences; experience within the healthcare system
Wan et al. (2023)	Australia	To investigate current dietary patterns and to explore barriers and facilitators to dietary management in South Asian immigrants	Indian	18	18 and over	13 males, 5 females	Mixed methods. Survey. In-depth semi-structured interviews	Challenges to adopting dietary changes; misconceptions about diet; social stigma; influence of family, friends, and community on diabetes management; governmental and organisational support
Waqar & Kuuire (2024)	Canada	To investigate perceptions and experiences of South Asians living with T2D in Ontario, and their utilisation of diabetes related services within the provincial healthcare system	Pakistani, Indian, Bangladeshi, Sri Lankan	20	36 – over 65	10 males, 10 females	In-depth interviews	Challenges with diet and physical activity; response to diagnosis; role of family doctor; lack of awareness about available resources; health insurance coverage

### Study characteristics

Eleven studies were conducted in the UK, three in Australia, two each in Norway, Canada and the USA, and one each in Denmark and New Zealand. Study participants were primarily of Indian, Pakistani or Bangladeshi origin. Two studies also focused on Sri Lankan and Nepalese migrants, while another study focused on Bhutanese refugees. The majority of studies included both female and male South Asian migrants. The ages of participants ranged from 18 to 84 years. Four of the studies were mixed-methods studies, while the remaining 18 were qualitative studies.

### Methodological quality

Among the qualitative studies, a detailed account of the underpinning philosophical orientation was not provided by more than half of the studies. Similarly, any specifications about the potential influence, including cultural influences, of researchers on the findings were not available in many studies. Five studies [62–66] have not specified the sampling strategy employed, and five studies [63–65, 67, 68] also did not specify the method of analysis used. Among the mixed-methods studies, two studies [1, 69] met 100% and 80% of the quality criteria for the qualitative and quantitative components, respectively, while one study met 80% and 100%, respectively [70]. The remaining study met only 60% of the quality criteria for both qualitative and quantitative components [71] (See Supplementary Material 2).

### Thematic analysis

The findings are presented as five key themes: (1) *Adapting to diabetes: impacts on food habits and dietary choices*; (2) *Diabetes threatens cultural conventions and identity*; (3) *Religious beliefs, practices, and prohibitions complicate diabetes management*; (4) *Social support and awareness to promote self-care*; and (5) *Individuals seek multiple sources for diabetes management* (Table 2).

**Table 2:** Key Themes and Sub-Themes from Thematic Analysis

Key theme		Sub-themes
1. Adapting to diabetes: impacts on food habits and dietary choices	1.1 Devise dietary strategies to cope with diabetes demands 1.2 Suggested dietary patterns are often viewed cumbersome 1.3 Healthy eating is a trade-off between ‘our food’, and ‘goray/Western’ food	
2. Diabetes threatens cultural conventions and identity	2.1 Cuisine symbolises culture and identity 2.2 “Diabetes defies cultural” identity of South Asians 2.3 Gender roles impede efficacy in diabetes management	
3. Religious beliefs, practices, and prohibitions complicate diabetes management	3.1 Common religious practices and prohibitions 3.2 Faith assures well-being	
4. Social support and awareness to promote self-care	4.1 Family and friends’ support is critical for self-care 4.2 Diabetes awareness facilitates effective management	
5. Individuals seek multiple sources for diabetes management	5.1 Perception of medication influences initiation and adherence 5.2 People sought traditional and complementary remedies	

### Adapting to diabetes: Impacts on food habits and dietary choices

This theme encompasses the dietary strategies individuals employ to manage their condition, the challenges they encounter when altering their usual dietary patterns, and the barriers to dietary modification. The theme is presented under three sub-themes.

#### *Devise dietary strategies to cope with diabetes demands*

The strategies used by South Asians to manage their condition after being diagnosed with T2D could range from more sustainable approaches to some drastic strategies. One sustainable and practical approach was to substitute sugars in tea with artificial sweeteners and full-fat milk with low-fat milk [72]. Similarly, some individuals removed ‘unhealthy’ ingredients such as sugar from their diet [72]. Another common strategy employed by South Asians was to consume their preferred food, including roti, curry and sweets, while limiting the portion size [72]. Others consumed Western food such as toast, sandwiches, cereals, soups and crisps for breakfast, lunch and snacks [72]. Some of the individuals followed a unique strategy to manage diabetes, which involved referring to blood glucose levels to manage dietary intake [1, 62].

*It [checking my glucose frequently] tells me whether I should eat or take some kind of snack or what... If there is any kind of high, then I avoid it, I try to bring it down by you know, not eating anything or eating healthy stuff like vegetables, or other stuff *[62]*.*

The underpinning motivators for some drastic approaches were gained through the experiences of individuals during religious fasting, such as Ramadan and *Karva chot* (a Hindu event, an annual fast observed by wives praying for their husbands to have a longer life) [72]. However, some individuals only made minor changes or no changes in their style of cooking as a dietary modification to manage diabetes. Healthy methods of cooking, such as baking and grilling, were seldom used, and any changes adopted were also tied to their traditional customs [65].

#### *Suggested dietary patterns are often viewed cumbersome*

Although South Asians adopted healthy dietary patterns, it was a constant struggle for them to maintain the changes adopted, especially during family and community gatherings and religious events [73]. For example, during religious events, some individuals avoided the dining area, believing it to be the only feasible strategy, though it impeded their social and community interactions [73].

Despite perceiving South Asian staple foods as a risk to effective diabetes management, some individuals could only adopt remedial changes [72]. In contrast, some individuals reported being careful and strict with their diet, though it was initially challenging for them [74]. Their struggle may be a result of their dependency on South Asian food, underpinned by habit and routine over several decades, and perceptions of their foods being ‘strength-giving foods’ [72, 74, 75] as noted below:

*In Pakistan they eat rice and chapattis all day, in every meal. You have to eat it, you can't get energy eating leaves and salad, you can't live off those things—our bodies ask for it or you won't be able to function* [75].

Their struggle was further exacerbated due to the perception that baked and boiled foods are tasteless, bland and unpalatable, and their unfamiliarity with the taste and methods of that style of cooking [65, 72]. South Asians believed such foods to be ‘lightweight’, and therefore felt their consumption would lead to feelings of lethargy. They further noted these feelings as a hindrance to fulfilling their duties and responsibilities towards others [72]. Nonetheless, they attempted to incorporate dietary changes by substituting or removing ‘unhealthy’ ingredients during food preparation. However, the etiquette of using communal pots for food preparation and serving impeded their attempts [72]. In addition, the perceived demands for additional time and expenditure in buying and preparing non-traditional foods further impeded their attempts at incorporating changes to their traditional dietary patterns [75].

#### *Healthy eating is a trade-off between ‘our food’, *and* ‘goray/Western’ food*

Individuals were observed to have a mixed understanding of healthy eating. Some believed that they should consume more boiled, baked and grilled foods, while others had the understanding that healthy eating is associated with consuming a balanced diet, which included carbohydrates, fruits and vegetables, but not meat [63, 72]. Among individuals, segregation was observed between the food they consumed and what Western people consumed, ‘our food’ and ‘goray food’, respectively. As Fagerli et al. [76] highlighted, some people believed that the preferred food for diabetes was bread, as they were receiving advice with a greater preference for ‘bread meals’. Interestingly, some people also believed that their South Asian diet was healthier, especially due to its filling nature and use of fresh ingredients and spices [75, 77].

Despite their perception of Western food and the created segregation, many South Asian migrants perceived their own food to be detrimental to diabetes management. This perception may be fuelled by the advice received from healthcare professionals to avoid some of ‘their food’ and from their observation that their host-country counterparts experienced lower rates of diabetes [72, 76]. As indicated by Fagerli et al. [76], such a devaluation of one’s culture’s foods and depiction of certain food cultures as ‘not good enough’ may impede effective diabetes management.

#### *Diabetes threatens cultural conventions and identity*

This theme identifies the cultural, symbolic and social significance South Asian migrants attach to ‘their food’, their constant struggle to retain their identities and enjoy ‘their food’, and how cultural family dynamics influence diabetes management, especially for women. The theme is presented under three sub-themes.

##### Cuisine symbolises culture and identity

South Asians displayed great importance and meaning to ‘their food’ in their daily lives. This was especially noticeable during conversations about family and community life in host countries. The symbolic and social meanings they attached to their cuisine and dietary practices were very similar despite the great diversity in ethnicity, religion, age and gender [72]. In religious events, special occasions such as weddings and celebrations of important life events, and when extending hospitality, South Asian food played a central role [72]. Thus, during food preparation, oil, ghee and certain other ingredients (spices, condiments, and sugar) were used more liberally, through which they signified affluence [65]. For South Asians, flavour and taste held prominence over presentation [65]. For many individuals, food was also a medium for bringing families and friends together [72, 75].

##### Diabetes defies cultural identity of South Asians

Most South Asians identify and hold their cuisine with cultural, symbolic and social meanings. Some felt that adopting dietary modifications demanded them to change *themselves*, which they were unable and unwilling to do [72, 76].

*Some things we cannot be disciplined about, we maybe eat too much of the wrong kinds of foods, like rice, but we cannot give that up *[72].

*We can't eat healthy in our households, eating salads won't save you from getting diabetes, since our life started on these foods, since childhood, we need spicy traditional food, we eat it daily* [75].

They further felt that to manage diabetes effectively, they were required to reject their food traditions and their identities. A more emotive expression of this was observed when one respondent placed his hand over his heart, sighed, and said, ‘*then it starts sinking in*’ [64]. Rejecting one’s food and food traditions, especially for South Asians, was extremely difficult as their food was perceived to be creating an identity of ‘we’ or ‘us’ [72].

*But the thing is roti is the foundation food for us, we are used to it now… *[72].

Among South Asians, acts of commensalism held great significance. Therefore, any changes to traditional acts of commensalism were perceived either as an insult, an offence or as being rude. As food is central in the lives of South Asians, it was often difficult to refuse to consume roti, sweets, tea, biscuits and other rich meals in order to manage diabetes. Despite understanding the impact of sugar on diabetes management, individuals were unable to avoid gifting sweets to others [1, 64, 65, 72, 73, 75, 78].

…*Important guests are entertained with snacks and sweets and rich meals. This is part of the hospitality code and refusal to partake is considered indecorous and unfriendly, and in certain contexts even insulting *[65].

Often, this meant that South Asian migrants would be faced with a choice of rejecting their traditions and identity, and consequently cause offence to others, or consuming food despite perceiving it as unfavourable for their diabetes management.

##### Gender roles impede efficacy in diabetes management

In addition to the common struggle of retaining one’s food traditions and identity while adopting healthy dietary habits, South Asian migrant women experienced more difficulties, as they fulfilled multiple roles, such as mother and wife [64, 74, 78] and were responsible for meal preparation, managing the household and looking after the needs of everyone in the family. There was a higher prominence set on the needs of the family than on their own needs, which complicated their diabetes management [72, 73, 78] as noted in the quote below:

…*if I’m too busy, then I will just eat what I’ve cooked for everybody, because I don’t have some time to make something for myself* [78].

Some women believed that men with diabetes received more family support in comparison to women with diabetes, especially from their partners [78]. However, some women also acknowledged that diabetes management could be more difficult for men as they generally bear the responsibility of earning for the family and thus have the additional pressure of paid work [78].

#### *Religious beliefs, practices, and prohibitions complicate diabetes management*

This theme highlights different practices and prohibitions South Asians follow depending on their religion, the influence of these on overall diabetes management, including acceptance towards medication and medication use, and the different faiths of individuals and their influence on diabetes management. The theme is presented under two sub-themes.

##### Common religious practices and prohibitions

Individuals, especially men who followed Islam, placed a great priority on fasting during Ramadan, regular attendance at prayers, and going on ‘Hajj’. Their belief that fasting is a religious obligation that cannot be missed had an impact on their diabetes management and overall health [37, 64]. Some South Asians also believed that religious fasting did not cause any harm to their diabetes condition and overall health. Thus, as highlighted below, some individuals altered the timings of medication and meals to align with their religious fasting [37, 71]:

*I take morning medicines in sehri (before sunrise) that is at 4 am and night medicines at 5:30 o’clock in iftar (after sunset). I am fasting from long time and even after diagnosed with diabetes...it is compulsory in Islam, I am doing because of my religion not for diabetes *[37]*.*

However, some individuals believed that fasting was harmful to diabetes management [37]. For example, Islam states that infants, pregnant women, lactating women and people who are ill and travelling are exempt from fasting during Ramadan [65]. As fasting creates difficulties in maintaining blood glucose levels, some individuals abstained from fasting on health grounds [37, 68] as noted in the quote below:

*Fasting is harmful for diabetic patients. I advise people not to fast. People fast because of fear and social pressure and not for religion. Many people I know fast just to show others *[37]*.*

Certain medication-taking behaviours, especially the uptake of insulin and other medicines that were thought to be derived from animals, were observed to be negatively influenced due to religious reasons. The principal reason for disapproval was that some religions, for example, Hinduism and Islam, do not approve of the intake of animal flesh or any other animal product [37, 70].

*If some medicines are made from animal sources especially pig then I would avoid taking it because it’s prohibited, Haram, in Islam *[37].

On the contrary, some individuals had positive views on the use of insulin derived from animals (such as pigs or cows) despite religious prohibitions, if insulin complements their diabetes management, as noted in the quote below:

*Cow and pork are prohibited in my religion (Hinduism) but if really evidence is there and support that it will work for my diabetes then I don’t care, I will take* [37].

##### Faith assures well-being

Similar to religious practices and prohibitions, the spiritual beliefs of individuals were also observed to influence their diabetes management, especially their medication-taking behaviours. For example, some believed that a ‘god’ or ‘a higher power’ looked after their health [62]. Despite their belief in a god or a higher power, they also believed that it is their responsibility to look after their health, as they have free will to decide for themselves.

…*the life is given by God …Allah wants me to maintain my body and keep taking care of my body ...If you get a disease, you need a medication. I mean it's not gonna happen that you are not gonna take the medication and say God will give me strength, that will not happen *[62].

Notably, some South Asians, especially those who followed Islam, were observed to believe that their diabetes was a test from Allah [71]. Therefore, diabetes was associated with their religious or spiritual fate. Some further believed that remaining faithful would help them manage their health [64, 69].

Praying regularly or receiving blessings from their religious leaders was believed by some South Asians to help manage diabetes. This was observed among those who followed Islam and Hinduism in particular. The perceived well-being due to praying and natural practices such as yoga was associated with their inner peace or ability to relieve stress, which eventually helped to manage blood glucose levels. Sometimes, praying was believed to provide strength to manage diabetes [37]. However, the power of blessings from religious leaders to manage their diabetes was purely dependent on the beliefs of an individual.

*Yes, I strongly believe in Islam. There are some powers there in Quranic verses that normally given by any Molana or Haz. I believe that some Dua (prayer) could resist some of your diseases including diabetes *[37].

Similarly, fasting, especially during Ramadan, was also observed to be associated with perceived well-being [68, 71]. Interestingly, some individuals also believed that the vegetarian diet they consumed due to religious prohibitions helped them manage diabetes [37].

*I am following Jainism, and I am pure vegetarian because Ahimsa (non-violence) principle encourages us (to) take vegetables. It helps in controlling diabetes *[37].

#### *Social support and awareness to promote self-care*

This theme highlights the significance of family and friends in facilitating diabetes self-management among individuals, the awareness of diabetes, and the importance of diabetes education in promoting effective self-management of diabetes. The theme is presented under two sub-themes.

##### Family and friends' support is critical for self-care

The family was observed as a supportive factor, either directly or indirectly, for diabetes management. Indirect support, especially for women, was in the form of support to complete household chores [78]. The direct support received varied from advice for an appropriate diet, and assistance for improved adherence to medication regimens, to facilitating communication with healthcare providers [62, 78]. Generally, individuals believed that close-knit families and living closely with the community provided them with social support that was valued and desired [66]. However, for some individuals, extended families were observed as less supportive and, at times, as a hindrance, especially when it came to incorporating and maintaining dietary changes [73, 78].

Friends, unlike family, were observed more as a source of shared experience [78]. Despite having a good individual understanding of diabetes and self-management behaviours, obtaining information from friends was perceived as beneficial [78]. Often, individuals received advice on managing diabetes from family and friends, including families living overseas, which at times complicated effective diabetes management [1, 63, 68, 77].

*My children told me not to fast… But then I called my big sister [who has type 2 diabetes] and she asked me if I fasted, and then I said no… and then my sister said ‘you should start, you will feel much better’. And it’s true, I do feel better when I fast* [68].

Sometimes the advice individuals received was in relation to a balanced diet and exercise, whilst other times the advice was on complementary approaches for diabetes management [63, 66]. Some individuals also believed that their diabetes management had a positive influence during visits to their home countries, simply by being close to family [77].

##### Diabetes awareness facilitates effective management

Some South Asians were aware that T2D is more prevalent within their communities and believed that their lifestyle and dietary habits were partly responsible for the development of diabetes in South Asians [72]. Interestingly, awareness about the causes of diabetes varied greatly among individuals, including reasons such as genetics, sugar in the blood, lifestyle, or that it was due to something caught during a visit to their home country [63]. Some believed diabetes was due to excessive stress, and others, to sweet consumption during the winter months in their host country [64].

Concerning diabetes management, South Asians recognised the importance of incorporating dietary changes, dietary management and leading an overall healthy lifestyle in managing their diabetes [72, 73, 75]. Interestingly, some individuals were not aware of the association between healthy eating and diabetes [63]. The understanding of some individuals resonated primarily around sugar as the reason for diabetes. For them, avoiding added sugar in their diet was the strategy for diabetes management [73]. There was limited awareness of recommended blood glucose levels. Only a few individuals were reported to have a good understanding of recommended levels and the potential complications of unregulated blood glucose levels [62, 72]. Awareness about the benefits of effective glycaemic control in preventing cardiovascular complications and improving quality of life was equally poor [69].

Furthermore, understanding and awareness about the available treatment options were also limited [69, 74]. Some individuals did not follow their medication regimen as they had a limited understanding of how the medication worked and its potential benefits [79]. Not surprisingly, some also associated the severity of their diabetes with the number of tablets they consumed, the use of insulin versus oral hypoglycaemics, and the number of hospital visits [64]. Despite this, many South Asians recognised the importance of health education, especially about diabetes, lifestyle modifications and medication [66, 73].

*We need diabetes education. We have no exercise here; the lifestyle is different here… What can we do to control diabetes? How can we avoid diabetes? We need information about diet. We want to understand how we can control diabetes *[66].

#### *Individuals seek multiple sources for diabetes management*

This theme identifies different perceptions of individuals towards oral anti-diabetic medication and insulin therapy, concerns associated with medications, and preferences for natural remedies, alternative and complementary approaches for diabetes management. The theme is presented under two sub-themes.

##### Perception of medication influences initiation and adherence

South Asian migrants generally had a belief that medications have side effects [37, 66, 67, 73, 79, 80]. Therefore, some deliberately attempted to reduce their intake of medications [67, 73] without consulting their healthcare professionals. Some individuals also reported consuming their medication only when their self-monitored blood glucose levels were high [67]. Several others avoided their medications, especially when travelling to their home countries for holidays [77]. Some tried to manage their diabetes purely through changes to their lifestyle [80]. The underpinning reasons for these behaviours were to avoid or mitigate the short-term side effects of their diabetes medications [67] and their dislike towards any long-term dependency on medications [80].

Some individuals expressed concerns over the health implications of consuming several medications together, as they believed the medications could counteract each other [81], as indicated in the quote below:

*Initially it was just two metformin a day, and then it was increased to four by the doctor. And then there’s blood pressure tablets to take and then aspirins and so on. So it all adds up and, y’know, if you take seven, eight pills a day and you wonder [laugh] is it the right thing? This can’t be good for me in the long run *[67].

Most individuals, however, adhered to oral anti-diabetic medications despite concerns, as they perceived them as important elements of their diabetes management [67, 79] and the medications provided an opportunity to be somewhat lenient on their diet and lifestyle behaviours.

Unlike oral medication, attitudes towards initiating insulin therapy were not positive. Some of the concerns highlighted by South Asians were anxiety and fear towards needles, the requirement of frequent injections, weight gain, maintaining privacy, impact on social life, social stigma, loss of independence and the concern that insulin contained animal derivatives [69, 79, 82]. Another concern, especially among young South Asians, was the difficulty in finding a life partner, as many would be hesitant to marry someone with diabetes, as highlighted below: 

*My mother told me that if I go on to insulin, I won’t be able to get married *[69].

Furthermore, insulin therapy was perceived to be an indication of a severe form of diabetes, which negatively influenced acceptance [69] as noted below:

*Insulin means you have a very bad form of diabetes, which can lead to heart problems and kidney failure. I have heard that insulin is a last resort *[69].

Despite these concerns, some individuals accepted the use of insulin, as they saw the suffering of family members due to poor glycaemic control [82].

##### People sought traditional and complementary remedies

Due to the pervasive concerns over the use of Western medication, some reported that they tried natural remedies that were commonly used in their home countries, or complementary and alternative medicines. For example, some South Asians consumed bitter foods, such as bitter gourd and bitter melon, canereed, and spices like cinnamon and fenugreek seeds, believing these foods help them to manage their blood glucose levels [62, 63, 66, 79, 80]. 

Some individuals preferred meditation and yoga as alternative and complementary approaches for their diabetes management [37, 73]. Some South Asians believed that meditation helped in controlling their blood glucose levels by strengthening their immune system [37].

*Yeah, meditation and yoga help at some point in time …I personally recommend having balance, doing both, like diet and exercise plus meditation. It really works well for me *[37].

## Discussion

This systematic review explores the influence of cultural and religious beliefs and practices on the self-management of T2D in South Asian migrants. The thematic analysis highlighted how South Asian migrants adapt to diabetes and its impact on their food habits, dietary choices, cultural conventions and identity. Further, it highlights how religious beliefs and practices, family care and community support may influence diabetes self-management. Finally, it emphasises that social support and community awareness of diabetes and medication adherence could promote self-management of diabetes for South Asian migrants.

### *Adapting to diabetes, threat to cultural conventions and identity*

Individual migrants attempt to manage diabetes by incorporating changes to their dietary choices and habits, but the adoption is often perceived to be cumbersome. Shah and colleagues [83] observed similar tendencies in their study population, who were Bangladeshi adults in the USA. Similar to South Asians, East Asians, such as Chinese migrants, also feel greatly challenged if they are to change their traditional dietary habits. This is because, similar to South Asian culture, rice and certain other foods that are high in carbohydrates are considered staples, sources of energy and comfort foods [84].

In certain cultures, food not only alleviates hunger but may also contribute to shaping the cultural and ethnic identities of individuals and communities [81]. It involves profound cultural symbolism and is crucial for an individual’s identity and integrity, as observed in the present review. Thus, individuals attach cultural, social, and symbolic meaning to their food. This is further supported by the findings of Vallianatos and Raine [85]. Retaining and maintaining traditional dietary patterns are believed to help migrants experience the feeling of being at home and maintain a familiar sense of self [15, 81, 85]. Any change to traditional patterns is often not welcomed, as they perceive these changes as threats to their familiar self. Therefore, refusing any potentially unhealthy food for the sake of diabetes management may be extremely difficult for South Asian migrants. Primarily due to the significance of food in their culture, family life, and events of socialising and celebration [83, 86]. 

Generally, it is plausible to think that following traditional practices involves more sweet, fried food, and high-fat dairy consumption, which may be detrimental to diabetes self-management. However, in the USA, despite following traditional cultural beliefs, South Asians were identified to associate with lower common carotid intima-media thickness [13]. This could be one of the factors influencing their initiative to engage in healthy behaviours. Therefore, it is imperative to reassess the interconnections between culture, health beliefs, dietary practices and health risks to shape diabetes self-management in this population.

In South Asian culture, culturally constructed and shaped gender roles for men and women as ‘providers’ for the family and ‘carers’ of the family, respectively, are not uncommon [86]. The traditional gender roles associated with food preparation and dietary management are, however, identified to be more pronounced only in certain cultures [87]. Generally, both roles prioritise family needs over individual needs, including health. The impact, however, may be more pronounced in women, as seen in the findings of the present study and the findings of Vallianatos and Raine [85], Li et al. [88], and Dimova et al. [87]. Since traditional gender roles are maintained in societies governed by social constructions and gender socialisation, women are inherently assumed to be caregivers in families [87, 89, 90]. This explains the tendency of men with diabetes to rely more on their spouses, especially for dietary management. Hence, the observation of men recognising their wives as a source of support, while women experienced less support from their husbands and families [91]. This is further evident by the findings of Ansari et al. [89], where Pakistani men view preparation of the prescribed diet and dietary management as a duty of the wives. A negative aspect of such culturally constructed and shaped gender roles may link with higher levels of perceived diabetes burden among women [92].

### *Religion and spirituality in diabetes management*

Religious beliefs and spirituality may play an important role in coping with diabetes; however, they may also have negative impacts on diabetes self-management. A common example may include Ramadan fasting in Islam. For individuals with diabetes, practising Ramadan fasting may include an increased risk of hypoglycaemic episodes and hyperglycaemic events, as well as an unbalanced nutrient intake and overeating [93, 94]. Individuals tend to fast despite these risks as they perceive fasting as an important obligation they are bound to fulfil. This is emphasised through the perspectives of T2D Muslim patients on Ramadan fasting in their study by Lee et al. [95]. Religious and spiritual influences may also be governed by a need to maintain an identity of their own with shared values and concerns, mediated by congregational attendance and faith and belief in god [13], interconnecting identity and religion. For example, in Islam, the five pillars of faith, of which Ramadan fasting is the fourth pillar (Sawm), define the basic identity of Muslim people, encompassing their faith, beliefs, and practices [96]. 

The negative influence of religion and spirituality on diabetes self-management may include fatalistic beliefs along with certain other beliefs [97]. Individuals may believe that a god or a higher power controls their health. This is emphasised in their study of South Asian migrants who suffered a first myocardial infarction by Dilla et al. [98] and in their study of cardiac patients by King-Shier et al. [99]. The present study also supports this observation, but also observes that South Asian migrants recognise the importance of being accountable for one’s own health and disease management despite any beliefs their religion may cultivate [62].

Similarly, religion and spirituality can facilitate positive cardiometabolic outcomes among South Asian migrants [100], possibly mediated by better mental health and coping outcomes [13]. Research discovered that people with diabetes may be prone to chronic stress due to their illness [101], and this stress is associated with increased anxiety and depression, poor glycaemic control and development of complications [102–104]. During such times, religiosity or spirituality can be a source of hope or strength for individuals with diabetes to better manage their condition [105]. Hence, engagement in prayers and receiving blessings from religious leaders. Religion and spiritual practices are, therefore, opportunities to direct targeted efforts to alleviate stress, anxiety, and potential depressive symptoms to improve compliance and achieve positive health outcomes. Such endeavours may be complemented by the involvement of respective religious leaders in designing and implementing programmes and interventions. For example, targeted pre-Ramadan education efforts to achieve improved metabolic outcomes, such as improved glycaemic control and body mass index (BMI) [94, 106].

### *Self-management through social support and alternative sources*

South Asians seek harmony between the intertwined culture, religion, and self-management, for which support from family, friends, relatives, and community is crucial. In South Asian culture, family and networks hold great significance [17]. Hence, individuals rely on their family and friends for support and advice when attempting to adopt and maintain diabetes self-management behaviours [98, 107]. Unlike in Western societies, in populations such as South Asian migrants, an individual is only a part or a node of a larger community. They become whole only when in harmony with their larger network [98]. This cohesion has health implications. For example, South Asian women living in the USA were observed to have lower BMI when in high social cohesion [108].

Social support should also encompass acceptance, encouragement of healthy behaviours and mitigation of stigmatisation. South Asian migrants may often fear being the subject of gossip – being stigmatised and considered ‘different’ – and isolation due to having diabetes [26]. Their experiences may be unique in comparison to host populations [109]. Such experiences may include getting labelled as a drug user (as insulin takes the form of an injection), being blamed or judged for having diabetes, being monitored, facing judgements from healthcare providers, and being unmarriageable [109–111]. 

In South Asian culture, the marriage prospects of young individuals may be severely affected due to a diagnosis of a chronic illness such as diabetes. A primary reason for this is the tradition of arranged marriage in South Asian cultures, where a marriage dowry from the bride’s side to the groom’s side is provided. The marriage dowry may create a considerable financial burden on the bride’s family as it takes the form of large sums of money, jewellery, properties or gifts. This burden may be exacerbated if the bride has a diagnosis such as diabetes [112]. Hence, lack of social support could lead to refusal of treatment advice and adherence to treatments to manage diabetes, which could subsequently lead to poor glycaemic control, complications, and sometimes death [110, 113]. Social support and psychological and physical well-being are, therefore, interconnected to each other and crucial in effective diabetes management [109].

To foster effective diabetes management in individuals, knowledge and understanding about diabetes, potential complications, effective self-management strategies, and the importance of glycaemic control and adherence to medication are equally crucial. Health literacy may mediate these understandings, and poor health literacy levels may hinder the ability of individuals to understand, apply and adhere to prescribed health advice and guidelines [114, 115]. Insulin derived from cows (bovine insulin) and pigs (porcine insulin) is no longer available commercially. The insulins in use are human insulin and analogues of human insulin, developed and produced through recombinant DNA technology [116]. However, misconceptions surrounding oral anti-diabetic medication and insulin may not be mitigated if individuals lack diabetes-related literacy. Health literacy could also mediate religion-cultivated prohibitions, practices and beliefs to support diabetes self-management. 

In addition, health literacy can support South Asian migrants to consciously choose between prescribed medication and alternative and complementary approaches for diabetes management. Similar to the observations of the present review, Chinese migrants may also strongly believe in traditional medicines, which may lead to individuals adhering less to prescribed Western medications or using traditional medicines concurrently with Western medications [28]. Thus, social support, health literacy, and religion may influence each other to mediate diabetes self-management.

In the lives of South Asian migrants, culture and religion create a complex web that is interconnected with identity, social support and health literacy, among many others. Hence, diabetes self-management for South Asian migrants cannot stand in isolation and demands navigation through a myriad of interconnected pathways.

###  Implications for practice, programme and resource development

Clinical practice improvements may accompany a multifaceted approach targeting the individual, family and friends, organisation (healthcare professionals), community and program development. Improvements may be initiated at the organisation level by training healthcare professionals to benefit all levels. Training should encourage understanding of the complexities surrounding South Asian migrants, including cultural and religious beliefs and practices, gender roles, the symbolic role of food, traditional dietary practices, the need for diabetes-related health literacy, and the complexities of shaping self-management in daily practice. Such an understanding would facilitate an effective dialogue between healthcare professionals and South Asian migrants and foster patient-centric, culturally safe, appropriate and respectful care for South Asian migrants. Furthermore, family and friends can be opportunities for action to improve diabetes self-management, especially by providing family-focused diabetes education. Through targeted education, stigma and misconceptions surrounding diabetes in the South Asian migrant community could also be addressed to facilitate community support for individuals. Thus, individuals would be able to effectively manage diabetes with the collective support of healthcare professionals, family and friends, which eventually would aid in improving the quality of life of individuals, averting diabetes-associated complications, and avoiding unwanted healthcare expenses. When designing resources and programmes for South Asian migrants, focus should be directed to reflect their values, beliefs, identities, cultures and diversities to facilitate better adoption. Care should also be taken in the approach to delivery, as South Asian migrants may often experience stigmatisation. Future research may explore more into how culture and religion shape self-management behaviours for diabetes, incorporating a more representative South Asian migrant sample.

### Strengths and limitations

To the best of our knowledge, this is the first systematic review exploring the influence of cultural and religious beliefs and practices on the self-management behaviours of South Asian migrants with T2D, incorporating a global perspective and evidence from studies with both qualitative and mixed-methods study designs. The majority of studies had a good overall methodological quality and were published in the last 10 years. However, the present exploration may not have captured certain beliefs and practices unique to Maldivian and Afghan migrants and their potential influences on diabetes self-management. Furthermore, the present review only considered peer-reviewed studies published in the English language, available in full-text, and retrieved only through the systematic literature search. Therefore, the retrieval may not include every study that addresses the area under consideration.

## Conclusions

This systematic review provides insight into the complex interrelationship of culture, religion, and T2D self-management in South Asian migrants, while recognising the importance of considering these influences as facets of multifaceted influences on an individual’s self-management behaviours rather than sole factors. Among South Asian migrants, traditional food and dietary practices, family and community support, traditionally constructed gender roles, stigma, religiosity and spirituality have significant impacts on the adoption and adherence to diabetes self-management behaviours. Thus, approaches to encourage the adoption of and adherence to self-management behaviours may accompany multifaceted approaches with a special focus on improving diabetes-related health literacy to facilitate improved self-management and patient-centric, culturally safe, appropriate and relevant care for South Asian migrants.

## Supplementary Information

Supplementary Material. Additional file 1: Systematic review database search strategies.

Supplementary Material. Additional file 2: Methodological quality assessment of studies included in the systematic review.

Supplementary Material. Additional file 3: Studies excluded from the systematic review.

Supplementary Material. Additional file 4: Data analysis canvas view from Quirkos Qualitative Data Analysis Software.

Supplementary Material. Additional file 5: PRISMA 2020 Checklist.

## Data Availability

All data generated or analysed during this study are included in this article.

## Abbreviations

CINAHL: Cumulative Index to Nursing and Allied Health Literature
BMI: Body Mass Index
MeSH: Medical Subject Headings
MMAT: Mixed Methods Appraisal Tool
PRISMA: Meta-Analyses
T1D: Type 1 diabetes
T2D: Type 2 diabetes
