# Supplementary material for: The influence of culture and religion on the self-management of type 2 diabetes in South Asian migrants: a systematic review
Source: BMC Public Health. 2026 Jun 5;26:2604. doi: 10.1186/s12889-026-27776-5 (PMC13579719; doi:10.1186/s12889-026-27776-5)
Supplement: Supplementary file 1 [file 12889_2026_27776_MOESM1_ESM.docx]

| 1 | exp Diabetes Mellitus, Type 2/ | 198500 |
| --- | --- | --- |
| 2 | ((type 2 or type II) adj3 diabet*).mp. | 286673 |
| 3 | (T2DM or T2D or NIDDM).tw. | 76202 |
| 4 | (Non insulin* depend* or noninsulin* depend* or Noninsulin?depend*).mp. | 12767 |
| 5 | 1 or 2 or 3 or 4 | 291853 |
| 6 | Self-Management/ or Self Care/ | 45418 |
| 7 | (Self-management* or self management* or selfmanagement* or self care).mp. | 84552 |
| 8 | Diet, Healthy/ or Diet/ | 214352 |
| 9 | (healthy eat* or diet* or eating habit* or dietary habit*).mp. | 1023887 |
| 10 | Diet* behavio?r*.mp. | 6615 |
| 11 | Exercise/ or (exercis* or physical* activ*).mp. | 640916 |
| 12 | Medication Adherence/ | 22132 |
| 13 | (medication adherence* or treatment adherence* or medication compliance*).mp. | 50974 |
| 14 | ((monitor* or control*) adj3 (blood sugar* or blood glucose* or glycemic* or glycaemic* or HbA1c or A1C)).mp. | 75101 |
| 15 | Life style/ or (lifestyle* or life-style* or life style*).mp. | 217359 |
| 16 | 6 or 7 or 8 or 9 or 10 or 11 or 12 or 13 or 14 or 15 | 1869436 |
| 17 | exp Asia, Southern/ | 198765 |
| 18 | (South Asia* or Southern Asia* or Maldives or Bangladesh or Bhutan or India or Afghanistan or Nepal or Pakistan or Sri Lanka).mp. | 327890 |
| 19 | 17 or 18 | 327973 |
| 20 | "Transients and Migrants"/ | 16219 |
| 21 | (migrant* or immigrant* or emigrant*).mp. | 70920 |
| 22 | ("Cultural* and linguistic*" or CALD).mp | 4438 |
| 23 | Cultural diversity/ or cultural* divers*.mp. | 18439 |
| 24 | Ethnicity/ or ethnic*.mp. | 262824 |
| 25 | Refugees/ or Refugee*.mp. | 22568 |
| 26 | 20 or 21 or 22 or 23 or 24 or 25 | 354785 |
| 27 | 5 and 16 and 19 | 2361 |
| 28 | 26 and 27 | 376 |

**Supplementary Material 1.** Systematic review database search strategies

**Medline (Ovid)**

**Embase Classic+Embase (Ovid)**

| 1 | exp non insulin dependent diabetes mellitus/ | 457898 |
| --- | --- | --- |
| 2 | ((type 2 or type II) adj3 diabet*).mp. | 381747 |
| 3 | (T2DM or T2D or NIDDM).tw. | 129028 |
| 4 | (Non insulin* depend* or noninsulin* depend* or Noninsulin?depend*).mp. | 442446 |
| 5 | 1 or 2 or 3 or 4 | 531522 |
| 6 | self care/ | 98733 |
| 7 | (Self-management* or self management* or selfmanagement* or self care).mp. | 124599 |
| 8 | diet/ or healthy diet/ | 342146 |
| 9 | eating habit/ | 19447 |
| 10 | (healthy eat* or diet* or eating habit* or dietary habit*).mp. | 1499473 |
| 11 | Diet* behavio?r*.mp. | 8558 |
| 12 | physical activity/ | 301388 |
| 13 | exercise/ | 478494 |
| 14 | (exercis* or physical* activ*).mp. | 1093224 |
| 15 | medication compliance/ | 63203 |
| 16 | (medication adherence* or treatment adherence* or medication compliance*).mp. | 93906 |
| 17 | ((monitor* or control*) adj3 (blood sugar* or blood glucose* or glycemic* or glycaemic* or HbA1c or A1C)).mp. | 169512 |
| 18 | lifestyle/ | 170462 |
| 19 | (lifestyle* or life-style* or life style*).mp. | 372552 |
| 20 | 6 or 7 or 8 or 9 or 10 or 11 or 12 or 13 or 14 or 15 or 16 or 17 or 18 or 19 | 2902593 |
| 21 | exp south asia/ | 336910 |
| 22 | (South Asia* or Southern Asia* or Maldives or Bangladesh or Bhutan or India or Afghanistan or Nepal or Pakistan or Sri Lanka).mp. | 498232 |
| 23 | 21 or 22 | 508072 |
| 24 | migrant/ | 18675 |
| 25 | immigrant/ | 24223 |
| 26 | emigrant/ | 611 |
| 27 | (migrant* or immigrant* or emigrant*).mp. | 81724 |
| 28 | ("Cultural* and linguistic*" or CALD).mp. | 5966 |
| 29 | cultural diversity/ | 4914 |
| 30 | cultural* divers*.mp. | 10162 |
| 31 | ethnicity/ | 167261 |
| 32 | ethnic*.mp. | 451312 |
| 33 | refugee/ | 20910 |
| 34 | Refugee*.mp. | 25937 |
| 35 | 24 or 25 or 26 or 27 or 28 or 29 or 30 or 31 or 32 or 33 or 34 | 548222 |
| 36 | 5 and 20 and 23 | 5678 |
| 37 | 35 and 36 | 779 |

**CINAHL (EBSCO)**

| S46 | (S44 AND S45) | 188 |
| --- | --- | --- |
| S45 | (S5 AND S23 AND S33) | 914 |
| S44 | (S34 OR S35 OR S36 OR S37 OR S38 OR S39 OR S40 OR S41 OR S42 OR S43) | 170748 |
| S43 | "cultural* divers*" | 21219 |
| S42 | (MH "Cultural Diversity") OR (MH "Cultural Values") | 28903 |
| S41 | "Cultural* and linguistic*" or CALD | 2548 |
| S40 | refugee* | 13149 |
| S39 | (MH "Refugees") | 10742 |
| S38 | ethnic* | 105859 |
| S37 | (MH "Ethnic Groups") | 41192 |
| S36 | migrant* or immigrant* or emigrant* | 35787 |
| S35 | (MH "Immigrants") | 19185 |
| S34 | (MH "Transients and Migrants") | 9 |
| S33 | (S24 OR S25 OR S26 OR S27 OR S28 OR S29 OR S30 OR S31 OR S32) | 100678 |
| S32 | Maldives or Bangladesh or Bhutan or India or Afghanistan or Nepal or Pakistan or "Sri Lanka" or "South Asia" or "Southern Asia" | 95547 |
| S31 | (MH "Sri Lanka") | 2566 |
| S30 | (MH "Pakistan") | 7243 |
| S29 | (MH "Nepal") | 3926 |
| S28 | (MH "Afghanistan") | 2876 |
| S27 | (MH "India") | 54490 |
| S26 | (MH "Bhutan") | 368 |
| S25 | (MH "Bangladesh") | 5853 |
| S24 | South Asia* | 7797 |
| S23 | (S6 OR S7 OR S8 OR S9 OR S10 OR S11 OR S12 OR S13 OR S14 OR S15 OR S16 OR S17 OR S18 OR S19 OR S20 OR S21 OR S22) | 742376 |
| S22 | "life#style*" | 63346 |
| S21 | (MH "Life Style Changes") OR (MH "Life Style") | 52044 |
| S20 | (monitor* or control*) N3 ("blood sugar*" or "blood glucose*" or glycemic* or glycaemic* or HbA1c or A1C) | 37937 |
| S19 | (MH "Glycemic Control") | 16156 |
| S18 | (MH "Blood Glucose Self-Monitoring") | 4705 |
| S17 | "medication adherence*" or "treatment adherence*" or "medication compliance*" | 33510 |
| S16 | (MH "Medication Compliance") | 27018 |
| S15 | exercis* or "physical* activ*" | 317734 |
| S14 | (MH "Physical Activity") | 63985 |
| S13 | (MH "Exercise") | 63698 |
| S12 | diet* behavio#r* | 8918 |
| S11 | "healthy eat*" or diet* or "eating habit*" or "dietary habit*" | 312786 |
| S10 | (MH "Food Habits") | 19551 |
| S9 | (MH "Diet") | 68507 |
| S8 | (MH "Diabetic Diet") OR (MH "Restricted Diet") | 6301 |
| S7 | self#management* or "self care" | 67513 |
| S6 | (MH "Self-Management") OR (MH "Self Care") | 55274 |
| S5 | (S1 OR S2 OR S3 OR S4) | 100796 |
| S4 | ("Non insulin* depend*" or "noninsulin* depend*" or Noninsulin#depend*) | 1601 |
| S3 | TI (T2DM or T2D or NIDDM) OR AB(T2DM or T2D or NIDDM) | 16814 |
| S2 | ("type 2" or "type II") N3 diabet* | 99562 |
| S1 | (MH "Diabetes Mellitus, Type 2") | 75474 |

**Web of Science (Clarivate)**

| 21 | #19 AND #20 | 504 |
| --- | --- | --- |
| 20 | #5 AND #12 AND #13 | 3567 |
| 19 | #14 OR #15 OR #16 OR #17 OR #18 | 677279 |
| 18 | TS=(Refugee*) | 59189 |
| 17 | TS=(ethnic*) | 378427 |
| 16 | TS=(cultural* divers*) | 97441 |
| 15 | TS=("Cultural* and linguistic*" or CALD) | 8649 |
| 14 | TS=(migrant* or immigrant* or emigrant*) | 204205 |
| 13 | TS=("South Asia*" or "Southern Asia*" or Maldives or Bangladesh or Bhutan or India or Afghanistan or Nepal or Pakistan or "Sri Lanka") | 708332 |
| 12 | #6 OR #7 OR #8 OR #9 OR #10 OR #11 | 4877785 |
| 11 | TS=(lifestyle* or life-style* or "Life style*") | 259291 |
| 10 | TS=("monitor*" or "control*" NEAR/3 "blood sugar*" or "blood glucose*" or glycemic* or glycaemic* or HbA1c or A1C) | 2636271 |
| 9 | TS=("medication adherence*" or "treatment adherence*" or "medication compliance*") | 46482 |
| 8 | TS=(exercis* or "physical* activ*") | 951657 |
| 7 | TS=("healthy eat*" or diet* or "eating habit*" or "dietary habit*" or "diet* behavio$r*") | 1291677 |
| 6 | TS=(Self-management* or "self management*" or "self$management*" or "self care") | 87613 |
| 5 | #1 OR #2 OR #3 OR #4 | 440282 |
| 4 | TS=("Non insulin* depend*" or "noninsulin* depend*" or "Noninsulin$depend*") | 13341 |
| 3 | TS=(T2DM or T2D or NIDDM) | 87908 |
| 2 | TS=("type 2" OR "type II" NEAR/3 diabet*) | 421159 |
| 1 | TS=("Type 2 Diabetes Mellitus") | 105167 |

**Scopus**

( ( TITLE-ABS-KEY ( "Type 2 Diabetes Mellitus"  OR  t2dm  OR  t2d  OR  niddm  OR  "Noninsulin*?depend*" )  OR  TITLE-ABS-KEY ( ( "type 2"  OR  "type II" )  W/3  diabet* ) )  AND  ( TITLE-ABS-KEY ( "Self?management*"  OR  "self care"  OR  "healthy eat*"  OR  diet*  OR  "eating habit*"  OR  "dietary habit*"  OR  exercis*  OR  "physical* activ*"  OR  "medication adherence*"  OR  "treatment adherence*"  OR  "medication compliance*"  OR  "life?style*" )  OR  TITLE-ABS-KEY ( ( monitor*  OR  control* )  W/3  ( "blood sugar*"  OR  "blood glucose*"  OR  glycemic*  OR  glycaemic*  OR  hba1c  OR  a1c ) ) )  AND  ( TITLE-ABS-KEY ( "South Asia*"  OR  "Southern Asia*"  OR  maldives  OR  bangladesh  OR  bhutan  OR  india  OR  afghanistan  OR  nepal  OR  pakistan  OR  "Sri Lanka" ) ) )  AND  ( TITLE-ABS-KEY ( "Transient* and Migrant*" OR migrant*  OR  immigrant*  OR  emigrant*  OR  "Cultural* and linguistic*"  OR  cald  OR  "cultural* divers*"  OR ethnic* OR refugee*  ) )

470
