# Supplementary material for: The influence of culture and religion on the self-management of type 2 diabetes in South Asian migrants: a systematic review
Source: BMC Public Health. 2026 Jun 5;26:2604. doi: 10.1186/s12889-026-27776-5 (PMC13579719; doi:10.1186/s12889-026-27776-5)
Supplement: Supplementary file 2 [file 12889_2026_27776_MOESM2_ESM.docx]

**Supplementary Material 2.** Methodological quality assessment of studies included in the review

**Table 1.** Methodological quality assessment of qualitative studies included in the review against the qualitative studies category of MMAT

| **#** | **Criteria** | Ahmad et al. (2021) | Ahmad et al. (2022) | Akhtar et al. (2024) | Choudhury et al. (2009) | Fagerli et al. (2005) | Iqbal et al. (2023) | Jamil et al. (2022) | Lawton et al. (2005) | Lawton et al. (2008) | Macaden & Clarke (2006) | Majeed-Ariss et al. (2015) | MuMin Chowdhury et al. (2000) | Mygind et al. (2013) | Patel et al. (2015) | Patel et al. (2016) | Timsina et al. (2022) | Uppal et al. (2016) | Waqar & Kuuire (2024) |
| --- | --- | --- | --- | --- | --- | --- | --- | --- | --- | --- | --- | --- | --- | --- | --- | --- | --- | --- | --- |
| 1 | Is the qualitative approach appropriate to answer the research question? | Yes | Yes | Yes | Yes | Yes | Yes | Yes | Yes | Yes | Yes | Yes | Yes | Yes | Yes | Yes | Yes | Yes | Yes |
| 2 | Are the qualitative data collection methods adequate to address the research question? | Yes | Yes | Yes | Yes | Yes | Yes | Yes | Yes | Yes | Yes | Yes | Yes | Yes | Yes | Yes | Yes | Yes | Yes |
| 3 | Are the findings adequately derived from the data? | Yes | Yes | Yes | Yes | Yes | Yes | Yes | Yes | Yes | Yes | Yes | Yes | Yes | Yes | Yes | Yes | Yes | Yes |
| 4 | Is the interpretation of results sufficiently substantiated by data? | Yes | Yes | Yes | Yes | Yes | Yes | Yes | Yes | Yes | Yes | Yes | Can’t tell | Yes | Yes | Yes | Yes | Yes | Yes |
| 5 | Is there coherence between qualitative data sources, collection, analysis and interpretation? | Yes | Yes | Yes | Yes | Yes | Yes | Yes | Yes | Yes | Yes | Yes | Yes | Yes | Yes | Yes | Yes | Yes | Yes |

**Table 2.** Methodological quality assessment of mixed-methods studies included in the review against the mixed methods category of MMAT

| **#** | **Criteria** | Abuelmagd et al. (2018) | Khan et al. (2008) | Naeem (2003) | Wan et al. (2023) |
| --- | --- | --- | --- | --- | --- |
| 1 | Is there an adequate rationale for using a mixed methods design to address the research question? | Yes | Yes | No | Yes |
| 2 | Are the different components of the study effectively integrated to answer the research question? | Yes | Yes | Yes | Yes |
| 3 | Are the outputs of the integration of qualitative and quantitative components adequately interpreted? | Yes | Yes | Yes | Yes |
| 4 | Are divergences and inconsistencies between quantitative and qualitative results adequately addressed? | Yes | Yes | Yes | Yes |
| 5 | Do the different components of the study adhere to the quality criteria of each tradition of the methods involved? | Yes | Yes | Can’t tell | Yes |
