# Supplementary material for: The influence of culture and religion on the self-management of type 2 diabetes in South Asian migrants: a systematic review
Source: BMC Public Health. 2026 Jun 5;26:2604. doi: 10.1186/s12889-026-27776-5 (PMC13579719; doi:10.1186/s12889-026-27776-5)
Supplement: Supplementary file 3 [file 12889_2026_27776_MOESM3_ESM.docx]

**Supplementary Material 3.** Studies excluded from the systematic review

| **Studies excluded** | **Reasons for exclusion** |
| --- | --- |
| Ahmad et al. (2022) | Does not focus on the influence of different aspects of culture and religion on T2D self-management behaviours discussed. |
| Gill (2012) | An abstract. Not a peer-reviewed full-text journal article. |
| Gucciardi et al. (2013) | Does not focus on the influence of different aspects of culture and religion on T2D self-management behaviours discussed. |
| Hyman et al (2017) | Does not focus on the influence of different aspects of culture and religion on T2D self-management behaviours discussed. |
| Khunti et al (2011) | A published report. Not a peer-reviewed full-text journal article. |
| Patel et al. (2012) | An abstract. Not a peer-reviewed full-text journal article. |
