# Supplementary material for: The influence of culture and religion on the self-management of type 2 diabetes in South Asian migrants: a systematic review
Source: BMC Public Health. 2026 Jun 5;26:2604. doi: 10.1186/s12889-026-27776-5 (PMC13579719; doi:10.1186/s12889-026-27776-5)
Supplement: Supplementary file 4 [file 12889_2026_27776_MOESM4_ESM.docx]

**Supplementary Material 4.** Data analysis canvas view from Quirkos Qualitative Data Analysis Software
